# PAD-Beads enrichment enhances detection of PrP^Sc^ using real-time quaking-induced conversion

**DOI:** 10.1186/s13104-019-4842-7

**Published:** 2019-12-13

**Authors:** Soyoun Hwang, Rohana P. Dassanayake, Eric M. Nicholson

**Affiliations:** 1 Virus and Prion Research Unit, National Animal Disease Center, United States Department of Agriculture, Agricultural Research Service, Ames, IA 50010 USA; 2 Ruminant Disease and Immunology Research Unit, United States Department of Agriculture, Agricultural Research Service, Ames, IA 50010 USA

**Keywords:** Scrapie, Prion diseases, RT-QuIC, Transmissible spongiform encephalopathy, PAD-Beads, Magnetic particle extraction

## Abstract

**Objective:**

Scrapie is a transmissible spongiform encephalopathy (TSE) that naturally occurs in sheep and goats. This fatal neurodegenerative disease results from misfolding of the normal cellular prion protein (PrP^C^) to a pathogenic prion protein form (PrP^Sc^). This pathogenic form, PrP^Sc^, accumulates in the brain and lymphoid tissues. The presence of PrP^Sc^ can be detected by an in vitro conversion assay known as real-time quaking induced conversion (RT-QuIC). RT-QuIC has been used to detect PrP^Sc^ in a variety of biological tissues from brains to fluids. While this technique is both rapid and sensitive, enhancing the detection of prions would be valuable in the diagnostic laboratories.

**Results:**

In this study, we assessed whether PrP^Sc^ detection sensitivity of RT-QuIC can be increased by enriching PrP^Sc^ in scrapie tissue homogenates using commercially available aggregated protein binding ligands coated magnetic beads (PAD-Beads). Coupling of RT-QuIC to PAD-Beads based cleanup allowed detection of PrP^Sc^ rapidly and without dilution of scrapie sheep brain homogenates prior to RT-QuIC. The PAD-Beads sample pretreatment step prior to RT-QuIC is a useful enhancement in the diagnosis of TSEs.

## Introduction

Mammalian prion diseases include human Creutzfeldt–Jakob disease (CJD), bovine spongiform encephalopathy (BSE), sheep scrapie, and cervid chronic wasting disease (CWD). These diseases are fatal neurologic diseases known as transmissible spongiform encephalopathies (TSEs), and they result from the misfolding of the normal cellular prion protein (PrP^C^) into a pathogenic form (PrP^Sc^) that accumulates primarily in the central nervous system [1–4].

Rodent bioassays can be considered a definitive technique for specific detection of prion infectivity. Two in vitro detection techniques such as protein misfolding cyclic amplification (PMCA) and real-time quaking-induced conversion (RT-QuIC) have demonstrated improved prion detection in PrP^Sc^ infected tissues. Both techniques are based on in vitro cell-free amplification of misfolded proteins present in samples. PMCA assay has been useful for detection of PrP^Sc^ in various samples including tissue and biological fluid samples of human and animals [5–8], but it requires specifically prepared normal animal brain material as a substrate and conversion is not observed in real-time. In contrast, recombinant prion proteins (rPrP^C^) are used as substrates for RT-QuIC reactions and RT-QuIC monitors the fibril formation in real-time [9–12]. RT-QuIC assay has been applied to the detection of a variety of sample types including cerebrospinal fluid (CSF) [4, 12–15], blood [5–7], saliva [8] and nasal brushings [16, 17]. While RT-QuIC itself offers considerable improvements in sensitivity over non-amplification approaches such as western blot, enzyme-linked immunosorbent assay (ELISA), and immunohistochemistry, any pretreatment that would improve speed and sensitivity should be useful in RT-QuIC assay.

Protocols for prion purification allows us to detect and characterize prion in a complex mixture with inhibitors and/or in a diluted samples to improve PrP^Sc^ detection sensitivity. Several studies have reported enhanced detection using PMCA and RT-QuIC assays by enrichment of PrP^Sc^ containing samples of various means such as immunoprecipitation [13], detergent enrichment [14], or non-specific metal surface binding [15, 16]. In this study, we have applied a commercially available sample enrichment kit called PAD-Beads that is based on a proprietary ligand that specifically binds misfolded proteins and allows for enrichment of PrP^Sc^ prior to detection and applied the approach to detection of PrP^Sc^ from scrapie-infected sheep brain samples by RT-QuIC.

## Main text

### Materials and methods

#### Source of tissue samples

All tissues samples used in this manuscript are from previously published sheep scrapie pathogenesis studies [17, 18] conducted at the National Animal Disease Center under the approval of the Institutional Animal Care and Use Committee (protocol number: ARS-2017-629). The animal experiments were carried out in accordance with the Guide for the Care and Use of Laboratory Animal (Institute of Laboratory Animal Resources, National Academy of Sciences, Washington DC).

#### PAD-Beads enrichment

A kit of PAD-Beads was purchased from Microsens Biotechnologies, London, UK [19, 20]. Samples were treated as described by the manufacturer. For elution, 12.5, 25, or 50 µl of 0.1 M NaOH, 0.1% Triton X-100, depending on desired final volume, was added to the beads and the tubes were shaken for 5 min. Tubes were placed on a magnet to capture the beads. While the tubes were on the magnet, the same volume, 12.5, 25, or 50 µl of 0.1 M HCl was added to the tubes to neutralize the alkali. Finally, liquid was removed and analyzed by RT-QuIC.

#### Recombinant prion protein production and purification

*E. coli* (BL21(λDE3)) was transformed with the pET28a vector containing the Met109 variant of bank vole PrP gene (amino acids 23–231; GenBank accession number AF367624) and the recombinant bank vole prion proteins were expressed and purified as described by Vrentas et al. [21, 22]. The concentration of protein is determined based upon UV absorbance at 280 nm using an extinction coefficient of 62005 M^−1^ cm^−1^ as calculated for the bank vole prion protein.

#### RT-QuIC protocol

RT-QuIC reactions were performed and analyzed as previously described [23–25]. The reaction substrate (98 µl) was composed of 10 mM phosphate buffer (pH 7.4), 300 mM NaCl, 0.1 mg/ml recombinant bank vole prion protein, 10 µM thioflavin T (ThT), 1 mM ethylenediaminetetraacetic acid tetrasodium salt (EDTA) and seeded with 2 µl of the indicated sample. ThT fluorescence measurements (excitation, 460 nm; emission 480 nm, bottom read, 20 flashes per well, manual gain 1400) were taken every 45 min with the reaction held at 42 °C in a BMG FLUOstar Omega plate reader for 100 h.

### Results

#### RT-QuIC detection of PAD-Beads captured scrapie prions

**Fig. 1:**
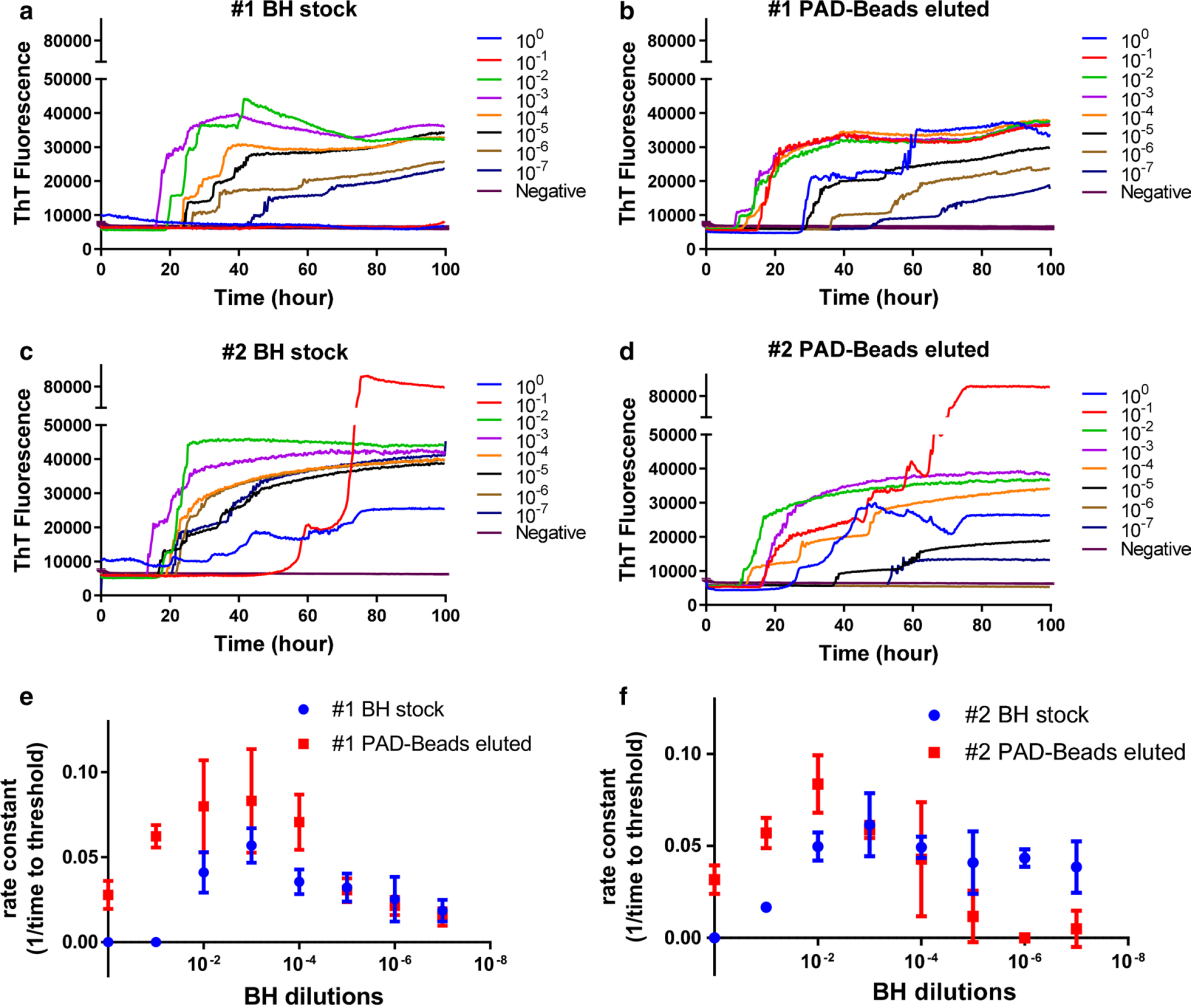
Comparison of RT-QuIC reactions between scrapie infected sheep brain homogenate (**a**, **c**) and PAD-Beads treated scrapie infected sheep brain homogenate (**b**, **d**). Comparison of rate constant obtained RT-QuIC reactions between scrapie infected sheep brain homogenate (**e**) and PAD-Beads treated scrapie infected sheep brain homogenate (**f**). RT-QuIC reactions were run using full-length (23–231) bank vole prion protein as the substrate with the addition of 0.001% SDS in the presence of 300 mM NaCl. Data are presented as mean ThT fluorescence of 4 technical replicates

To evaluate the efficacy of PAD-Beads based enrichment for the purposes of RT-QuIC, reactions containing recombinant bank vole prion protein (BV rPrP^C^) were seeded with different dilutions of PAD-Beads eluate for comparison with that of the directly diluted sheep brain homogenate and monitored for increased ThT fluorescence. RT-QuIC reactions containing bank vole substrate and 300 mM NaCl were seeded with brain stock solution (Fig. 1a, c) or the PAD-Beads eluate (Fig. 1b,d) and dilutions from 10^−1^ to 10^−7^ of brain homogenate from two sheep (#1 and #2) positive for scrapie. Both reactions seeded with brain stock and PAD-Beads eluted brain showed fibril formations as monitored by ThT fluorescence. Assays seeded with negative sheep brain homogenate did not produce ThT fluorescence under the conditions of the experiment. Assays seeded with high concentrations brain homogenate (non-diluted or 10^−1^ dilution) of brain homogenate also did not show ThT increase a result typically interpreted as indicative of inhibitors in the sample. In contrast, non-diluted PAD-Beads eluate seeded reactions exhibited positive ThT fluorescence suggesting PAD-Beads enrichment removes these unidentified inhibitors. Reactions seeded with PAD-Beads eluted brain samples showed a shorter lag time compared to the reactions seeded with non-PAD-Beads treated samples. A 20 h lag time was observed for non-PAD-Beads enriched brain homogenates dilutions from 10^−2^ to 10^−4^, but that lag time is reduced to only 10 h following enrichment with PAD-Beads for dilutions from 10^−1^ to 10^−4^. The undiluted PAD-Beads eluate without dilution showed the ThT fluorescence lag time of around 30 h. As can be seen in Fig. 1e, f, reaction assays seeded with PAD-Beads eluate brain dilutions between 10^0^ to 10^−4^ for animal # 1 and between 10^0^ to 10^−2^ for animal #2 show higher rate constants compared to the reactions seeded with brain homogenate dilutions.

#### Scrapie negative brain homogenate inhibits RT-QuIC detection of PAD-Beads eluted scrapie prions

**Fig. 2:**
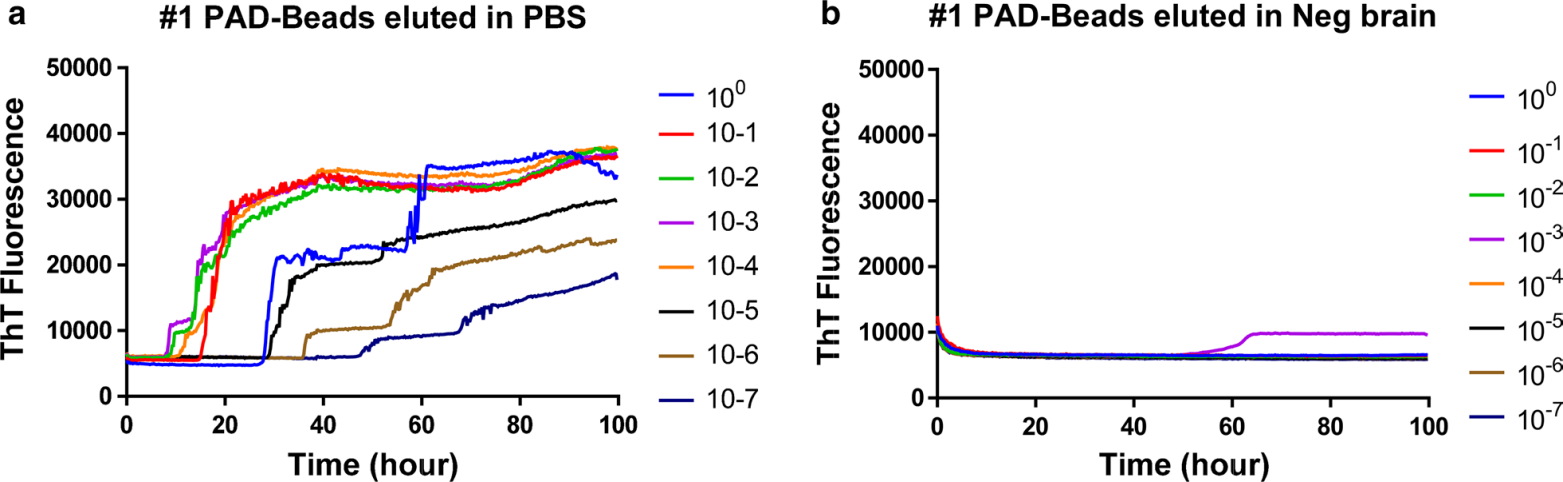
Comparison of RT-QuIC reactions seeded with PAD-Beads treated brain homogenate diluted in PBS (**a**) and PAD-Beads treated brain homogenate diluted in negative sheep brain homogenate (**b**)

In order to investigate the effect of inhibitory compounds that may be present in brain homogenate, PAD-Beads eluate of scrapie positive samples were diluted into 5% scrapie negative sheep brain homogenate. Unlike dilutions in PBS, RT-QuIC reactions did not produce any ThT increase from any assays with dilutions in scrapie-negative sheep brain homogenate confirming the presence of some inhibitory compounds (Fig. 2).

#### Effect of PAD-Beads elution volume on RT-QuIC

**Fig. 3:**
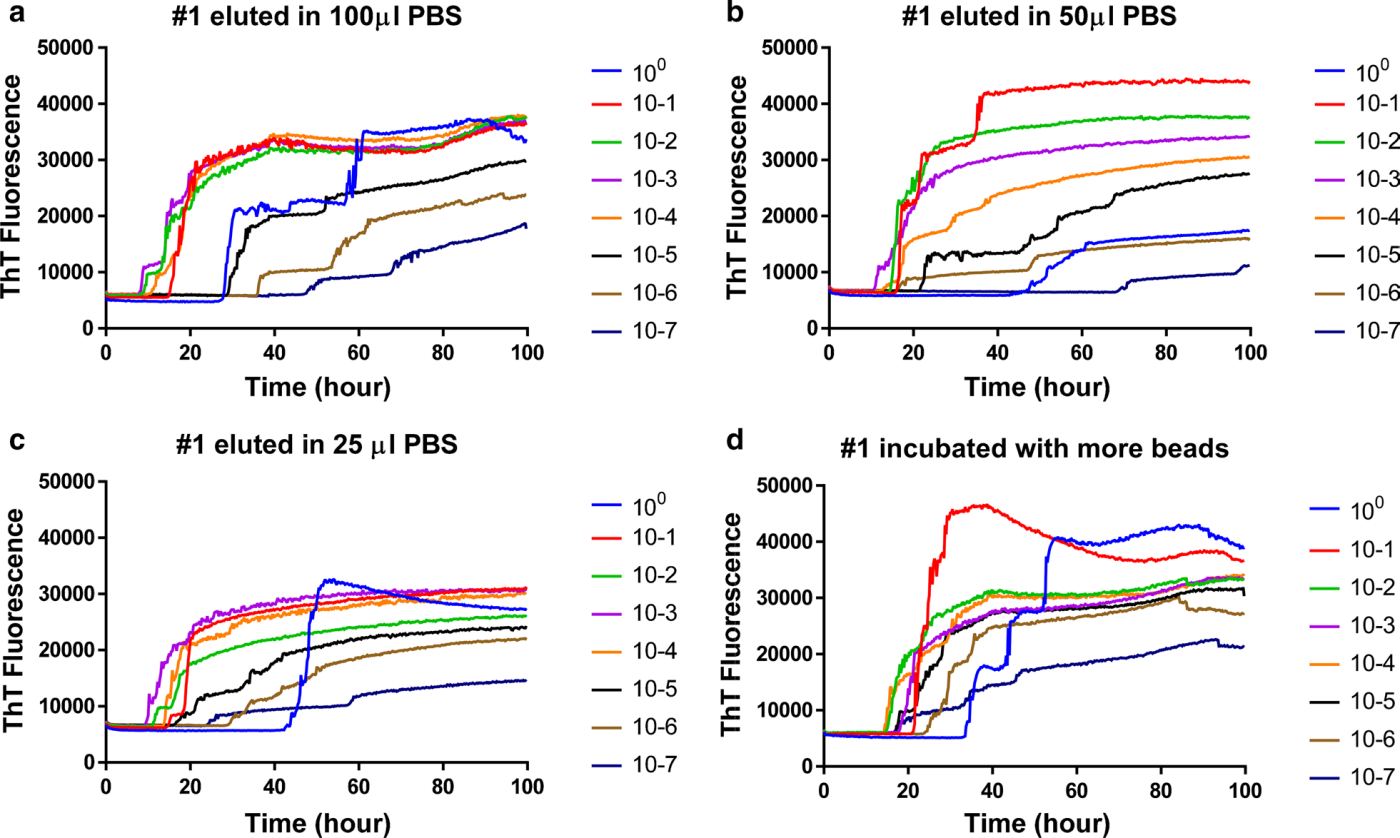
Comparison of RT-QuIC reactions seeded with different volume of elution buffer and PAD-Beads solution. All RT-QuIC reactions were run using full-length (23–231) bank vole prion protein as the substrate with the addition of 0.001% SDS in the presence of 300 mM NaCl. Data are presented as mean ThT fluorescence of 4 technical replicates

To assess PAD-Beads PrP^Sc^ binding capacity and efficiency, we also tested if a lower volume of elution buffer increases seeding activity. Compared to brain eluted in 100 µl PBS, brain sample eluted in 50 µl or 25 µl elution buffer showed faster seeding activity especially for higher dilutions like 10^−5^ or 10^−6^ as can be seen in Fig. 3. Reactions seeded with 10^−5^ or 10^−6^ dilutions of PAD-Beads eluted with 100 µl elution buffer started ThT increase at 40 and 50 h (Fig. 3a) but shorten to 20 and 30 h with a 50 and 25 µl volumes (Fig. 3b, c). Lower elution volume resulted in higher sensitivity likely due to higher concentration of PrP^Sc^ in the final elution.

#### Test of PAD-Beads binding capacity using RT-QuIC

To assess the PrP^Sc^ binding capacity of PAD-Beads, we used double the volume of PAD-Beads and eluted at the same (100 µl) volume. The use of a larger starting volume (200 µl) of PAD-Beads resulted in a reduced lag time (Fig. 3d). RT-QuIC reactions seeded with PAD-Beads eluted in a 100 µl volume but bound to twice the volume of PAD-Beads used in the standard protocol provided a similar reduction in lag time to the onset of ThT fluorescence to that observed for the standard elution volume. In this case, all brain dilutions up to 10^−7^ have higher seeding activity showing lag time shorter than 40 h compared to the reactions seeded with eluent from the lower amount of beads. Increased bead volume resulted in higher sensitivity indicating that for these samples and the lower amount of beads used the binding capacity of the beads was exceeded.

### Discussion and conclusions

RT-QuIC is an efficient tool to detect PrP^Sc^ from humans and animals [9–12, 26–28], even in the presymptomatic stage of disease [24, 29, 30]. With the amplification of sensitivity afforded by the RT-QuIC technique, early diagnosis is becoming an increasingly feasible proposition since the technique amplifies the pathogenic form of prion protein in biological tissue or fluid samples. In this study, we have shown that PAD-Beads sample enrichment enhances RT-QuIC detection of prions, likely through removal of inhibitors to amplification present in brain samples. Specifically, PAD-Beads enrichment shortens the time required to detect PrP^Sc^ by RT-QuIC by approximately 50% relative to unenriched samples.

Other approaches exist for enrichment of PrP^Sc^ prior to detection. For example, several studies showed that infectious prion adheres to stainless steel [16, 31–33], which allows enrichment of PrP^Sc^ through non-specific binding. Phosphotungstic acid precipitation of PrP^Sc^ is often applied prior to detection by PMCA and RT-QuIC [29, 34–37]. Most similar to the work presented here, a study by Orru et al. demonstrated that anti-PrP^Sc^ antibody (15B3) coated magnetic beads could bind infectious prions in brain and blood samples and prions bound to the beads could be detected by RT-QuIC [13]. Later, Denkers et al. coupled RT-QuIC detection to an iron oxide magnetic extraction method (IOME) with samples from brain, saliva, urine, faces, and cerebral spinal fluid (CSF) [15]. Collectively these studies show that enrichment of PrP^Sc^ relative to other components in the complex mixture that is present in a tissue homogenate enhance the detection of PrP^**Sc**^ by RT-QuIC, most likely due to the removal an undetermined contaminant that is inhibitory to the in vitro conversion process.

Commercially available PAD-Beads are a practical technique for separating misfolded proteins such as PrP^Sc^ from a complex mixture such as tissue homogenate or biological fluids. For the detection of scrapie prion using PAD-Beads, we have applied RT-QuIC and confirmed the presence of PrP^Sc^ by high seeding activity. PAD-Beads enrichment takes about 1 h, and the enriched samples resulted in a shorter lag time by approximately 10 h relative to unenriched samples for the detection of scrapie prions in brain samples. In addition, PAD-Beads enriched samples eluted with lower volume allowed higher detection sensitivity of RT-QuIC reactions. In a similar way, use of a larger starting volume (200 µl) of PAD-Beads shortened the lag time for detection of scrapie PrP^Sc^ by RT-QuIC.

Given the rapid-simple protocol and improved sensitivity shown here for detection of scrapie, the established ability of PAD-Beads to bind many different proteins in an amyloid conformation as well as the applicability of RT-QuIC to other amyloid diseases, this method could provide a useful enhancement to RT-QuIC for other diseases such as Parkinson’s Disease since RT-QuIC has now been applied successfully to amplify α-synuclein aggregates [38, 39].

## Limitations

The primary limitation of this work is that we did not specifically address additional TSEs, but we would anticipate that the method is applicable to other TSEs from other host species based upon the broad applicability of both PAD-Beads and RT-QuIC to various TSE agents. However, we cannot comment as to the degree of enhancement in sensitivity or the time savings. Despite these limitations, PAD-Beads enrichment does provide a useful method to enhance RT-QuIC diagnosis of scrapie.

## Data Availability

The datasets used and/or analyzed during the current study are in the manuscript and any additional details will be made available from the corresponding author on reasonable request.

## Abbreviations

TSE: transmissible spongiform encephalopathy
PrPC: normal cellular prion protein
PrPSc: pathogenic prion protein
RT-QuIC: real-time quaking induced conversion
CJD: Creutzfeldt–Jakob disease
BSE: bovine spongiform encephalopathy
CWD: cervid chronic wasting disease
PMCA: protein misfolding cyclic amplification
rPrPC: recombinant prion protein
CSF: cerebrospinal fluid
ELISA: enzyme-linked immunosorbent assay
ThT: thioflavin T
EDTA: ethylenediaminetetraacetic acid
BV: bank vole
